# Acquired resistance to molecularly targeted therapies for cancer

**DOI:** 10.20517/cdr.2024.189

**Published:** 2025-06-05

**Authors:** Nolan M. Stubbs, Tyler J. Roady, Maximilian P. Schwermann, Elias O. Eteshola, William J. MacDonald, Connor Purcell, Dinara Ryspayeva, Nataliia Verovkina, Vida Tajiknia, Maryam Ghandali, Viva Voong, Alexis J. Lannigan, Alexander G. Raufi, Sean Lawler, Sheldon L. Holder, Benedito A. Carneiro, Liang Cheng, Howard P. Safran, Stephanie L. Graff, Don S. Dizon, Sendurai A. Mani, Attila A. Seyhan, Robert W. Sobol, Eric T. Wong, Clark C. Chen, Ziya Gokaslan, Martin S. Taylor, Brian M. Rivers, Wafik S. El-Deiry

**Affiliations:** ^1^Cancer Health Equity Institute, Morehouse School of Medicine, Atlanta, GA 30310, USA.; ^2^Legorreta Cancer Center, Brown University, Providence, RI 02903, USA.; ^3^Hematology and Oncology, Brown University and Brown University Health, Providence, RI 02906, USA.; ^4^Department of Pathology and Laboratory Medicine, Brown University, Providence, RI 02912, USA.; ^5^Department of Neurology, Brown University, Providence, RI 02906, USA.; ^6^Department of Neurosurgery, Brown University, Providence, RI 02906, USA.

**Keywords:** Molecular targeted therapies, acquired resistance, cancer treatment strategies, precision medicine

## Abstract

Acquired resistance to molecularly targeted therapies remains a formidable challenge in the treatment of cancer, despite significant advancements over the last several decades. We critically evaluate the evolving landscape of resistance mechanisms to targeted cancer therapies, with a focus on the genetic, molecular, and environmental contributors across a variety of malignancies. Intrinsic mechanisms such as mutations, drug and drug target modifications, and, notably, the activation of the mitogen-activated protein kinase (MAPK) and phosphoinositide 3-kinase (PI3K)/Akt pathways are mechanisms different malignancies use to combat therapeutic effectiveness. Furthermore, extrinsic alterations to the tumor microenvironment contribute to therapeutic resistance. We highlight similarities and differences in mechanisms across a wide spectrum of cancers including hematologic malignancies, non-small cell lung cancer, gastrointestinal, breast, and prostate cancers, pancreatic, ovarian, endometrial, and intracranial gliomas. Emerging strategies to overcome resistance, including multi-targeted approaches, combination therapies, and exploitation of synthetic lethality, are all critically discussed. We advocate for a nuanced understanding of resistance mechanisms as a cornerstone for developing future therapeutic strategies, emphasizing the necessity for integrated approaches that encompass genomic insights and precision medicine to outpace the dynamic and complex nature of cancer evolution and therapy resistance.

## INTRODUCTION

### Global cancer burden and epidemiological trends

Cancer ranks as a leading cause of disease-related mortality across the globe. Per projections, this burden is estimated to increase by 47% in 20 years^[1]^. This alarming trend is hypothesized to be driven by an aging population, an increasing amount of exposure to environmental carcinogens, evolving dietary patterns, physical inactivity, and rising obesity rates in both high-income and low- to middle-income countries (LMICs)^[1,2]^. Importantly, the distribution of cancer types and risk factors varies considerably across geographic regions. This is reflected via the differences in healthcare infrastructure, screening programs, and prevalence of infectious agents associated with malignancies [i.e., hepatitis B virus (HBV) and human papillomavirus (HPV)]^[1]^.

Disparities in access to targeted therapies and precision oncology also contribute to global inequalities in cancer outcomes. While molecularly targeted therapies (MTTs) have revolutionized treatment paradigms in well-resourced settings, patients in resource-limited regions often lack access to genetic testing required to identify actionable mutations. This results in delayed diagnosis, inappropriate treatment selection, and poorer survival outcomes. Therefore, discussions of acquired resistance in targeted therapies must account for global treatment gaps, acknowledging that resistance dynamics in high-resource settings - where molecular profiling is routine - may differ significantly from those in settings where empirical therapy is the norm^[3,4]^.

As MTTs become more affordable and accessible globally, understanding the interplay between tumor biology, treatment access, and socio-environmental factors will be crucial for designing resistance-prevention strategies that are relevant across diverse populations^[1,3,4]^. This globalized perspective not only enhances scientific relevance but also aligns with the broader goals of reducing global cancer health disparities through the equitable application of precision medicine. All that being said, it is imperative for researchers and scientists alike to formulate and implement effective treatment approaches for patients with cancer^[1,2]^.

### Precision oncology and MTTs

Since their initial clinical endorsement in the late 1990s, MTTs have shown pronounced anticancer capabilities for both specific cancers and across cancer types. These therapies, which include small molecule inhibitors and therapeutic monoclonal antibodies that block signal transduction, have become integral to precision oncology. In 2001, the US Food and Drug Administration (FDA) approved imatinib, the first small-molecule targeted drug to be authorized for clinical use against chronic myeloid leukemia (CML)^[3,4]^. Since then, there has been a dramatic increase in the production of drugs. There are currently 89 small-molecule targeted anticancer drugs that have been approved by the US FDA and National Medical Products Administration (NMPA) of China^[5]^. An overview of representative MTTs including their mechanisms of action, associated resistance pathways, and clinical indications is provided in Table 1. While this has dramatically changed the way cancer is treated, the challenge of acquired resistance has sparked an ongoing struggle to understand how to effectively manage tumor growth.

**Table 1:** Overview of MTTs and resistance mechanisms

**Drug**	**Class**	**Target(s)**	**Mechanism of action**	**Resistance mechanisms**	**Tumor type(s)**
Vincristine	Vinca alkaloid	Tubulin	Inhibits microtubule formation → mitotic arrest	P-gp overexpression, tubulin mutations	Leukemia, lymphoma
Trastuzumab	Monoclonal antibody	HER2	Blocks HER2 dimerization and signaling, induces ADCC	HER2 mutations, pathway bypass, TME effects	Breast cancer
Ponatinib	TKI	BCR-ABL1 (incl. T315I)	ATP-binding site inhibition	Compound mutations, efflux, microenvironment interactions	CML, Ph+ ALL
Imatinib	TKI	BCR-ABL1	Competitive ATP-binding site inhibition	BCR-ABL mutations (esp. T315I)	CML, Ph+ ALL
Bosutinib	TKI	BCR-ABL1	ATP-binding site inhibition	BCR-ABL mutations, off-target toxicity	CML
Asciminib	STAMP inhibitor	BCR-ABL1 (Myristoyl pocket)	Allosteric inhibition	Emergent compound mutations	CML
Blinatumomab	Bispecific antibody	CD19	T-cell engagement and cytotoxicity	CD19 downregulation, lineage switch	B-ALL (including Ph+ ALL)
TMZ	Alkylating agent	DNA	DNA methylation at O6-guanine	MGMT overexpression	Glioblastoma
Bevacizumab	Monoclonal antibody	VEGF	Neutralizes VEGF	Alternate angiogenesis pathways	Glioblastoma, colorectal cancer, NSCLC
Erlotinib	TKI	EGFR	ATP-competitive EGFR inhibition	EGFR T790M mutation, MET amplification	NSCLC
Osimertinib	TKI	EGFR (T790M)	Irreversible covalent inhibition	EGFR C797S mutation, bypass pathways	NSCLC
Gefitinib	TKI	EGFR	ATP-competitive EGFR inhibition	EGFR T790M mutation, MET amplification	NSCLC
Crizotinib	TKI	ALK, MET	ATP-competitive inhibition	ALK resistance mutations (L1196M)	NSCLC
Ceritinib	TKI	ALK	ATP-competitive inhibition	ALK mutations, bypass signaling	NSCLC
Lorlatinib	TKI	ALK	ATP-competitive inhibition	Complex ALK mutations, off-target effects	NSCLC
Vemurafenib	TKI	BRAF V600E	Selective mutant BRAF inhibition	MEK reactivation, alternate splicing	Melanoma
Dabrafenib	TKI	BRAF V600E	Selective mutant BRAF inhibition	MEK reactivation, alternate splicing	Melanoma
Trametinib	MEK inhibitor	MEK1/2	Inhibits MEK kinase activity	Secondary MEK mutations, pathway reactivation	Melanoma
Sunitinib	TKI	VEGFR, PDGFR	Multikinase inhibition	Hypoxia-induced resistance, alternative angiogenesis	Renal cell carcinoma, GIST
Sorafenib	TKI	RAF, VEGFR, PDGFR	Multikinase inhibition	Adaptive hypoxia response	Hepatocellular carcinoma, renal cell carcinoma
Olaparib	PARP inhibitor	PARP1/2	Inhibits DNA repair	HR proficiency, replication fork protection	OvCa, breast cancer
Niraparib	PARP inhibitor	PARP1/2	Inhibits DNA repair	HR proficiency, replication fork protection	OvCa
Rucaparib	PARP inhibitor	PARP1/2	Inhibits DNA repair	HR proficiency, replication fork protection	OvCa
Pembrolizumab	Checkpoint inhibitor	PD-1	Blocks PD-1/PD-L1 interaction	Immunoediting, loss of MHC expression	Melanoma, NSCLC
Nivolumab	Checkpoint inhibitor	PD-1	Blocks PD-1/PD-L1 interaction	Immunoediting, loss of MHC expression	Melanoma, NSCLC

This table summarizes key molecularly targeted cancer therapies, their drug class, specific targets, mechanism of action, known resistance mechanisms, and associated tumor types. Targeted agents include TKIs, monoclonal antibodies, immune checkpoint inhibitors, and other therapeutic classes designed to disrupt oncogenic signaling. Resistance mechanisms are categorized based on genetic mutations (e.g., BCR-ABL1 mutations in chronic myeloid leukemia), pathway reactivation (e.g., MEK reactivation in BRAF-mutant melanoma), and alterations in the TME (e.g., hypoxia-induced resistance in renal cell carcinoma). Understanding these mechanisms provides insights into strategies for overcoming resistance and improving therapeutic efficacy. MTTs: Molecularly targeted therapies; HER2: human epidermal growth factor receptor 2; ADCC: antibody-dependent cellular cytotoxicity; TME: tumor microenvironment; TKI: tyrosine kinase inhibitor; ATP: adenosine triphosphate; CML: chronic myeloid leukemia; ALL: acute lymphocytic leukemia; STAMP: specifically targeting the ABL myristoyl pocket; TMZ: temozolomide; MGMT: methyltransferase; VEGF: vascular endothelial growth factor; NSCLC: non-small cell lung carcinoma; MET: mesenchymal-epithelial transition factor; ALK: anaplastic lymphoma kinase; MEK: mitogen-activated protein kinase kinase; PARP: poly (ADP-ribose) polymerase; OvCa: ovarian cancer; HR: homologous recombination; PD-1: programmed cell death protein 1; PD-L1: programmed death-ligand 1; MHC: major histocompatibility complex.

## FOUNDATIONS OF THERAPEUTIC RESISTANCE

### Defining therapeutic resistance

Resistance to MTTs can be separated into two categories: Intrinsic (primary), where resistance mechanisms pre-exist prior to therapy initiation, or acquired, where therapy itself exerts selective pressure that promotes clonal evolution and survival of resistant subpopulations^[6]^. Acquired resistance often reflects a dynamic process involving genomic instability, epigenetic reprogramming, and interaction with the tumor microenvironment (TME), all of which promote a more treatment-refractory tumor phenotype^[7]^. Due to the evolutionary aspect of this concept and the difficulty encountered when attempting to address these changes, acquired resistance will remain the main focus of this manuscript.

The causes of acquired resistance are varied and can involve many different aspects including drug-target modifications, alternative signaling pathway activation, and/or shifts in the TME usually initiated by genetic adaptations^[8]^. Understanding the mechanisms surrounding different treatment failures is vital for improving patient outcomes, as this will help us develop new treatment strategies and therapeutic targets.

### Key pathways underpinning resistance dynamics

Before diving into the various mechanisms of resistance to MTTs, it is important to gain an understanding of signaling pathways that promote tumorigenesis. The landscape of cancer resistance mechanisms is significantly influenced by the reconfiguration of key cellular signaling pathways, notably the mitogen-activated protein kinase (MAPK) and the phosphoinositide 3-kinase–protein kinase B/Akt (PI3K-PKB/Akt) pathways^[9,10]^. These pathways play pivotal roles in the survival, proliferation, and therapy resistance observed across various cancer types. Thus, understanding how these pathways contribute to resistance is vital for the development of effective counterstrategies.

#### MAPK

**Figure 1:**
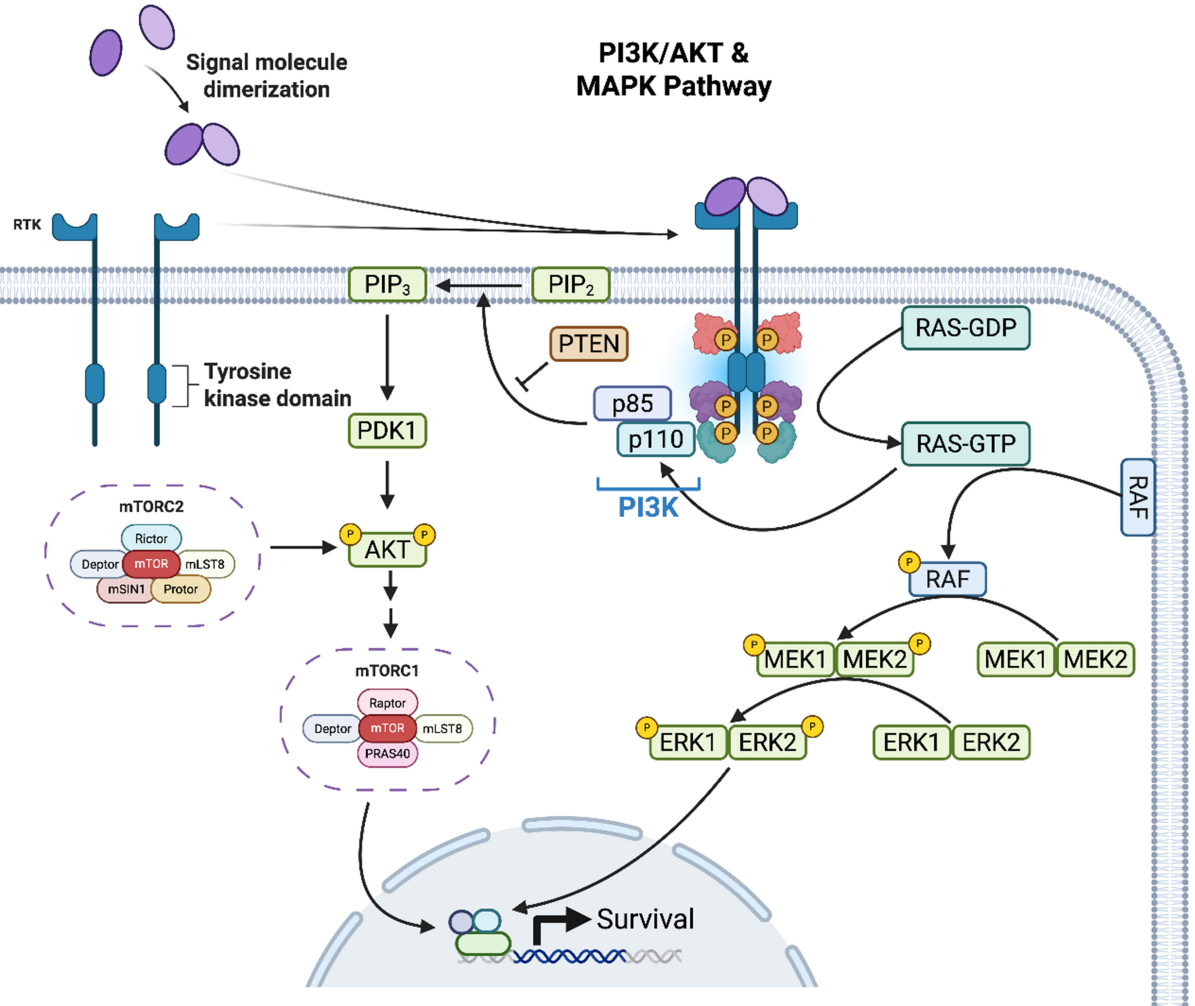
Schematic representation of parallels between PI3K/Akt and MAPK signal pathways. Both pathways become activated when growth factors bind to their respective RTKs, causing RAS GTPases to relay signals downstream. Activation of these pathways promotes increased cell proliferation and survival. Some of the intricacies in signaling by mTORC1 and mTORC2 are shown leading to some distinct effects on metabolism and cell fate. PI3K: Phosphoinositide 3-kinase; Akt: protein kinase B; MAPK: mitogen activated protein kinase; RTK: receptor tyrosine kinase; RAS: oncogene; PTEN: phosphatase and tensin homolog; PIP_2_: phosphatidylinositol 4,5-bisphosphate; PIP_3_: phosphatidylinositol 3,4,5-triphosphate; PDK1: 3-phosphoinositidedependent protein kinase 1; mTORC1: mTOR complex 1; mTORC2: mTOR complex 2; RAF: rapidly accelerated fibrosarcoma; MEK: mitogen-activated extracellular signal-regulated kinase; ERK: extracellular signal-related kinase. Created in BioRender. Purcell, C. (2025) https://BioRender.com/3us5vg.

The MAPK pathway is integral to the regulation of cell growth and survival, as shown in Figure 1. Activation of this pathway begins at the cell membrane with the binding of growth factors to their respective receptor tyrosine kinases (RTKs), which, in turn, activate their associated RAS GTPases to relay signals downstream^[11]^. RAF family kinases (ARAF, BRAF, CRAF) are the direct effectors of RAS, which phosphorylates and activates MEK, to then phosphorylate ERK, which translocates to the nucleus to regulate gene expression by activating transcription factors^[11]^. The linear progression from growth factor stimulation to transcriptional regulation underscores the MAPK pathway’s pivotal role in mediating cellular responses to external cues. The MAPK pathway also has crosstalk with other signaling pathways, including PI3K/Akt, where mTORC2 appears to convey a portion of the oncogenic Ras signal in melanoma and likely in other contexts^[11]^.

Mutations in any components of this pathway, particularly in RAS or RAF, can lead to its constitutive activation, driving oncogenesis by promoting uncontrolled cell proliferation and survival. For example, in melanoma, the BRAF V600E mutation results in constitutive activation of the MAPK pathway, promoting uncontrolled cell growth. Though BRAF inhibitors were initially impactful for melanoma, resistance, particularly to vemurafenib, eventually emerged, facilitated by secondary mutations reactivating the MAPK pathway. Much like the initial base switch mutation that causes permanent activation of BRAF, base switch mutations in MEK1 and MEK2 can also cause them to become aberrantly activated and continue to affect downstream pathways^[12]^. Other mechanisms known to cause resistance include overexpression and resultant amplification of the RAF kinases (BRAF, CRAF) along with downstream activating mutations in N-RAS, MEK1, or Akt1^[13-16]^.

#### PI3K/Akt

Parallel to the MAPK pathway, the PI3K/Akt pathway plays a crucial role in cancer cell metabolism, growth, and survival, as seen in Figure 1. This pathway’s activation also begins with the binding of growth factors to the extracellular regions of their respective RTKs and GPCRs^[17,18]^. Upon activation, RTKs recruit and activate the PI3K. Once turned on, these PI3Ks catalyze downstream reactions, which eventually recruit Akt to the plasma membrane for further mediators like 3-phosphoinositidedependent protein kinase 1 (PDK1) and mTOR complex 2 (mTORC2) to act on it, activate it, and facilitate a myriad of downstream targets to increase cell proliferation and survival^[17]^.

In discussing tumorigenesis, this pathway is constantly activated following either mutations in PIK3CA or the loss of the tumor suppressor PTEN, which acts as a key regulator of Akt activation^[19]^. When discussing melanoma and BRAF resistance, the PI3K pathway is often implicated as a bypass mechanism conferring survival following targeted therapy^[20,21]^. The interaction between the MAPK and PI3K/Akt pathways exemplifies the complexity of intracellular signaling networks and their role in cancer resistance. Crosstalk between these pathways allows cancer cells to maintain proliferative and survival signaling even when one pathway is pharmacologically inhibited. The scenario can be even more complicated by NRAS mutations, present in a notable subset of melanomas^[22]^. Alterations in NRAS lead to activation of the RAS-RAF-MAPK and PI3K-Akt pathways at similar levels seen with BRAF mutations^[22,23]^. Furthermore, NRAS mutations rarely occur with changes in the PI3K-Akt pathways, suggesting a dominant role for NRAS mutations in these signaling processes. As a result, there is a disruption in cell cycle regulation that promotes survival mechanisms and cellular proliferation^[22]^. As BRAF inhibitors are seemingly ineffective in these tumors, the development of downstream MEK inhibitors served the purpose of slowing growth in these tumors^[24,25]^. Unfortunately, however, MEK frequently mutates following MEKi treatment, resulting in either overactivation of MEK or the inability of the inhibitor to bind MEK^[26,27]^. This crosstalk is particularly evident as the mutations lead to concurrent activation of both pathways, presenting a significant challenge to single-targeted therapies.

#### EGF/EGFR

Beyond the PI3K/Akt and MAPK pathways, the EGF/EGFR signaling axis also plays a pivotal role in resistance development^[28]^. EGFR overexpression, activating mutations, and autocrine signaling loops contribute to both intrinsic and acquired resistance. Additionally, the tumor suppressor PTEN - an upstream regulator of the PI3K pathway - is frequently lost or mutated in resistant cancers, further amplifying downstream survival signaling^[29]^. The insulin-like growth factors (IGFs) and their receptors also play a role, driving pro-survival signals and metabolic reprogramming that enhances cancer cell fitness under therapeutic pressure^[30,31]^.

## THE TME AND THERAPY RESISTANCE

### Definition and core components of the TME

The interaction between cancer cells and their surrounding TME plays another important role in developing resistance to targeted therapies^[32]^. The TME is a supportive meshwork of biological components that aid in the growth and development of a tumor^[33-35]^. More specifically, these components are immune cells, stromal cells, extracellular matrix (ECM), extracellular vesicles (EVs), cytokines, and growth factors^[33-35]^. Collectively, these elements contribute significantly to therapeutic resistance through direct and indirect interactions with cancer cells.

### Myeloid cells and resistance

Tumor-associated macrophages (TAMs) originate from bone marrow and play an important role in the TME and subsequent therapeutic resistance^[36-38]^. Studies have shown that TAMs can directly induce epithelial-to-mesenchymal transition (EMT) of tumor cells and are heavily involved in ECM remodeling of the TME^[36-38]^. Activation of EMT induces stem cell properties in cancer cells^[36-38]^. Cancer cells with stem cell properties are known to be resistant to various treatments, including chemotherapy, targeted therapy, radiation, and immunotherapies. EMT inducers, such as transforming growth factor-β (TGF-β)^[39]^ and tumor necrosis factor-α (TNF-α), are secreted by TAMs along with proteases like cathepsins and matrix metalloproteinases (MMPs) to help to facilitate ECM degradation. Thus, there is subsequent enhanced tumor cell motility and therapeutic resistance. The role of TAMs also extends to promoting angiogenesis within the tumor tissue, which supports tumor growth and subsequent expansion.

Myeloid-derived suppressor cells (MDSCs) can also impact tumor therapeutic resistance by promoting an immunosuppressive environment within the TME^[40]^. For example, it has been shown that MDSCs secrete interleukin 10 (IL-10), which serves to inhibit macrophage activation and consequently reduces the secretion of immunogenic cytokines, thereby dampening intratumoral immunity^[41]^. This suppression of immune activity contributes to an environment where tumor cells can evade immune surveillance and resist therapeutic interventions.

### Stromal cells and resistance

Beyond myeloid cells, cancer-associated fibroblasts (CAFs) within the TME can also play a pivotal role in modulating cancer resistance^[42]^. Like TAMs and MDSCs, CAFs secrete proteins, exosomes, and factors involved in ECM remodeling^[42]^. Furthermore, these factors can influence tumor cells in a paracrine manner and activate signaling pathways such as Wnt/β-catenin, PI3K/Akt, and MAPK^[43-45]^. CAFs also secrete growth factors such as TGF-β, fibroblast growth factor (FGF), epidermal growth factor (EGF), and hepatocyte growth factor (HGF) to induce EMT-like CAMs and exacerbate the aggressiveness and resistance of tumor cells^[46]^.

### Role of EVs in therapy resistance

EVs are increasingly recognized as critical mediators of drug resistance in cancer^[47-49]^. These vesicles facilitate intercellular communication by transferring oncogenic proteins, drug-efflux pumps, and non-coding RNAs between cancer cells, thereby spreading resistance traits across the tumor population^[47-49]^. For example, EVs derived from resistant tumors have been shown to deliver P-glycoprotein to neighboring sensitive cells, promoting multidrug resistance^[47-49]^. Additionally, EVs play an important role in remodeling the TME, modulating immune evasion, and promoting angiogenesis, all of which contribute to the development of resistance^[50]^.

## ACQUIRED RESISTANCE MECHANISMS ACROSS CANCER TYPES

Following the preview of the underlying pathways that contribute to therapy resistance and the multifaceted role of the TME in fostering these resistances, we can now pivot our attention toward a comprehensive examination of acquired resistance mechanisms as they unfold across a spectrum of cancer types. Each cancer type presents unique challenges and elucidates the adaptive nature of cancer cells in the face of targeted therapies. This section aims to highlight the nuanced intricacies of resistance mechanisms, providing a foundation for the development of more effective, multifaceted treatment strategies that anticipate and counteract these adaptive responses.

### Genetic and molecular basis of resistance in hematologic malignancies

The battle against hematologic malignancies, particularly CML and acute lymphocytic leukemia (ALL), has been at the forefront of personalized medicine, driven by the advent of MTTs. However, the emergence of acquired resistance represents a formidable challenge, undermining the efficacy of these treatments and complicating patient management strategies.

#### CML and therapeutic resistance

In the context of CML, the main driver mutation is a t(9;22)(q34;q11) balanced reciprocal translocation event, which results in the formation of the Philadelphia chromosome characterized by a BCR:ABL1 fusion gene^[51]^. This gene encodes a constitutively active tyrosine kinase, resulting in an unchecked myeloproliferative state diagnostic of CML^[51]^. The Philadelphia chromosome, resulting from the t(9;22) translocation, occurs in approximately 25% of adult ALL cases and 2%-5% of pediatric ALL cases, significantly influencing treatment decisions and prognosis^[52]^. From this finding, the focus shifted to the manufacturing of tyrosine kinase inhibitors (TKIs) like imatinib, bosutinib, dasatinib, and nilotinib and helped to revolutionize CML treatment. Although not considered curative, they are effective for the long-term prevention of disease progression in a majority of individuals with life expectancy in patients with CML nearing that of the general population^[53-56]^. In chronic-phase CML, TKI therapy has achieved remarkable success, with 5-year overall survival rates exceeding 90%. In contrast, the prognosis for Ph+ ALL remains more guarded, with 5-year survival rates in relapsed or refractory cases below 50%, even with newer therapies^[53-56]^. Despite this progress, unfortunately, approximately 20% of patients develop resistance to first-line TKIs. This most commonly occurs through the development of point mutations in the catalytic domain of the BCR:ABL1 protein, rendering first-line agents ineffective^[53-56]^. The most famous and prominent resistance mutation is the T315I mutation, as it notoriously confers resistance to all first-line TKI treatments^[57-59]^. This results in the use of alternative, more toxic agents to slow the progression of disease.

Beyond the BCR:ABL1-specific forms of resistance, independent forms may be acquired through TKI-resistant leukemic stem cells, which can act as a reservoir for the propagation of new tumor cells once treatment stops^[59]^. Oftentimes, in BCR:ABL1-independent TKI-resistant CML, the RAF/MEK/ERK pathway will be activated through the increased expression of PKCη, whose subsequent phosphorylation of CRAF leads to increased proliferation and cell survival^[60]^. Resistance may also occur through the improper activation of the mTOR pathway, which induces aberrant autophagy that protects cells from TKI-induced apoptosis^[61]^. Both pathways may be activated irrespective of BCR:ABL1 signaling^[60,61]^. The aforementioned mechanism of resistance underlines the multifaceted nature of this disease, highlighting the necessity for comprehensive treatment approaches^[57-59]^. A recent breakthrough in CML management is asciminib, a first-in-class STAMP inhibitor targeting the ABL myristoyl pocket^[60]^. Unlike ATP-competitive TKIs, asciminib offers a novel mechanism that bypasses several common resistance mutations, including T315I. Clinical trials such as ASCEMBL have demonstrated superior efficacy and safety compared to bosutinib in resistant chronic-phase CML^[62,63]^.

#### ALL and therapeutic resistance

In a similar fashion to CML, ALL arises through the uncontrolled proliferation of lymphocyte precursor cells in the bone marrow and peripheral blood. Seventy-five percent of these cancers develop from precursors of the B cell lineage and the remaining twenty-five percent are derived from the T cell lineage^[64]^. Like many other hematologic malignancies, the genetic architecture of this disease is highly heterogeneous, with diverse mutations arising and frequent chromosomal translocation events even resulting in the occasional development of the Philadelphia chromosome much like CML^[65,66]^. In the case of Philadelphia chromosome-positive ALL, treatment with a TKI like imatinib is the current standard of care like CML, and thus, similar resistance mechanisms are likely to arise^[67,68]^. For Philadelphia chromosome-negative ALL, the highly efficacious treatment combination of anthracycline-like drugs, vincristine, and glucocorticoids (GC) results in complete remission for 80% of patients^[68]^. Vincristine, a vinca alkaloid chemotherapy agent, binds to tubulin, which inhibits microtubule formation during mitosis and eventually leads to cell cycle arrest in metaphase^[69]^. This mechanism makes it particularly effective against rapidly dividing cancer cells, such as those found in leukemias and lymphomas. Additionally, analogous to CML, the good prognosis of primary ALL is often overshadowed by dismal recurrence rates. In Ph+ ALL, ponatinib - a third-generation TKI - is particularly effective against the T315I mutation and is now considered a preferred frontline option in combination with reduced-intensity chemotherapy^[70-74]^. By binding the ATP-binding site of the BCR-ABL1 fusion protein, ponatinib blocks oncogenic signaling that drives proliferation and survival in Philadelphia chromosome-positive (Ph+) leukemias^[70-74]^. Its broad kinase inhibition profile also targets several off-target kinases, contributing to its efficacy, but also to its toxicity profile^[70-74]^. Furthermore, chemotherapy-free regimens combining ponatinib with blinatumomab, a CD19-targeting bispecific antibody, show promising efficacy in both frontline and relapsed settings^[75]^. Despite ponatinib’s potency, resistance can still emerge through compound mutations in BCR-ABL1, altered drug efflux, and leukemic microenvironment interactions^[74]^.

Anthracycline resistance is most commonly acquired through the increased expression of efflux ATP-binding cassettes (ABC)-transporters, reducing intracellular drug concentration^[76]^. For vincristine, there is evidence that resistance is acquired through mutations that can stabilize microtubules, offsetting the primary mechanism of therapeutic action^[69]^. Furthermore, resistance to GCs and subsequent relapse has been observed when mutations develop in the NR3C1 and BTG1 genes, which encode for the GC receptor and promote increased GC receptor expression, respectively^[77,78]^. BTG1 has been shown to stabilize glucocorticoid receptors (GR) by interfering with proteasome-mediated receptor degradation, thereby increasing receptor abundance and potentiating glucocorticoid signaling in lymphoid malignancies^[77]^. Like many cancers, increased RAF/MEK/ERK signaling has also been associated with relapse and GC resistance^[78]^.

### Pathway reconfiguration and drug resistance in solid tumors

Moving from the genetic and molecular landscape characterizing resistance in hematologic malignancies, the realm of solid tumors unfolds a distinct yet intricate narrative. Non-small cell lung carcinoma (NSCLC), gastrointestinal cancers, prostate cancer (PCa), breast cancer, ovarian cancer (OvCa), glioblastoma, and pancreatic cancer epitomize the sophisticated mechanisms through which solid tumors counteract targeted therapies.

#### NSCLC and tyrosine kinase

NSCLC serves as a primary case study in the adaptation against targeted treatments, especially against tumors harboring specific genetic markers like EGFR mutations and ALK rearrangements. NSCLC comprises up to 85% of lung cancer in the US, with development being due to driver mutations of different tyrosine kinases like KRAS, EGFR, ALK, ROS1, MET, RET, NTRK, human epidermal growth factor receptor 2 (HER2), and BRAF^[79,80]^. Much like what was discussed in CML, the discovery of these constitutively active receptors led to the development of molecular targeted therapies, with those against EGFR and ALK being the most efficacious^[81]^.

Despite the high initial response rates, NSCLC widely develops resistance through several proposed mechanisms, including on-target mutations, off-target mutations. On-target drug resistance occurs via a secondary mutation in the drug target. For instance, with first- and second-generation EGFR inhibitors, many EGFR-mutant NSCLC patients developed the T790M gatekeeper mutation, which hinders the drug from interacting with the kinase^[82]^. Analogously, the ALK mutation L1196M has been characterized as a gatekeeper mutation, preventing ALK inhibitors like crizotinib access to the kinase’s ATP binding site^[83]^. Though less common, another potential mechanism of on-target resistance is the amplification of the target kinase itself, which has been described for both EGFR and ALK^[84,85]^. The amplification of target genes, such as EGFR and ALK, further demonstrates how cancer cells can override the blockade established by targeted drugs, maintaining proliferative signaling despite treatment^[86,87]^. While direct modulation of treatment effect is paramount in TKI resistance in NSCLC, off-target mechanisms can essentially work in parallel to further diminish treatment efficacy following exposure to the drug. An example of such a mechanism is exemplified by the amplification of c-MET (an alternative tyrosine kinase), which can operate in parallel to EGFR and activate the same PI3K/Akt pathway, thereby negating the therapeutic benefit of EGFR inhibition^[86]^. This highlights the intricate web of signaling pathways within cancer cells and their inherent capacity to find alternate survival routes under therapeutic pressure.

#### Gastrointestinal carcinoma and 5-fluorouracil resistance

5-fluorouracil (5-FU) has been recognized as a cornerstone in therapeutic efficacy in the domain of gastrointestinal carcinoma (GIC)^[87]^. Unfortunately, therapeutic efficiency has diminished as resistance to this agent started to emerge. Mechanisms such as enhanced DNA repair mechanisms, shifts in drug metabolism, and the activation of survival pathways collectively forge a robust front against 5-FU.

Enhanced DNA repair, specifically through the base excision repair (BER) and mismatch repair (MMR) pathways, has been shown to play significant roles in promoting resistance to 5-FU^[88,89]^. The APC gene, identified as a BER-related protein, is notable in this context as tumors lacking functional APC exhibit resistance to 5-FU^[90]^. This occurs via the enzyme thymidylate synthase (TS), which functions as the target enzyme of 5-FU^[89]^. Overexpression is postulated to lead to reduced drug efficacy by providing an alternative pathway for DNA synthesis^[90,91]^. Dihydropyridine dehydrogenase (DPD) is another enzyme that plays a pivotal role in the catabolism of 5-FU, with its overexpression resulting in 5-FU degradation^[92,93]^. Furthermore, studies have demonstrated that tumors with deficient mismatch repair (dMMR) mechanisms are resistant due to an increased expression of TS and DPD, in comparison to proficient mismatch repair (pMMR) cells^[94]^. The enzymatic degradation of 5-FU via overexpressed DPD and the bypass mechanisms provided by elevated levels of TS reflect the dynamic interplay between drug efficacy and tumor survival strategies.

Activation of cellular survival pathways represents another dimension of 5-FU resistance. Notably, pathways such as PI3K/Akt, Wnt, and MAPK/ERK have been implicated in promoting cell survival and proliferation, enabling cancer cells to withstand the cytotoxic effects of 5-FU^[95-97]^. The activation of these pathways in response to 5-FU treatment underscores the adaptability of cancer cells, facilitating their survival in the face of therapeutic challenges. EMT has also been recognized as a key factor in 5-FU resistance^[98,99]^. In GIC, this process is associated with increased metastatic potential as it promotes tumor stemness and invasiveness.

Collectively, these mechanisms illustrate the intricate network of pathways contributing to 5-FU resistance in GIC. The insights garnered from understanding these resistance mechanisms are instrumental in the development of novel therapeutic strategies aimed at mitigating resistance and enhancing the efficacy of 5-FU-based treatments.

#### Breast carcinoma and resistance to HER-2 targeted therapies

When discussing difficult-to-treat solid tumors, it is essential that breast carcinoma (BC) is mentioned, as it is the most common malignancy affecting women in the United States^[100]^. HER-2-positive BC is a specific subtype of breast cancer characterized by overexpression of the HER2 protein^[101]^. The HER-2/neu (EGFR2 or ErbB2) is a transmembrane oncoprotein encoded by the *HER2/neu* gene and is a member of the family of RTKs that also includes EGFR (HER1, ErbB1), ErbB3, and ErbB4^[102]^. HER-2/neu is weakly detectable in epithelial cells of normal tissues but is overexpressed in approximately 20% to 25% of invasive breast cancers and has been linked to a poor prognosis and a high risk of cancer relapse^[103]^. Among FDA-approved HER2-targeted drugs are monoclonal antibodies (mAb), TKIs, and antibody-drug conjugates (ADC)^[103-105]^. Despite the benefits of anti-HER2 therapies in the survival rate of HER2 BC patients, unfortunately, it too follows the overarching theme of this manuscript.

Resistance is often acquired following initial treatment, with some patients being non-responsive from the start despite having the mutation of interest^[102,103,106-108]^. Acquired resistance to trastuzumab is often encountered in metastatic BC, making the search for clinically relevant mechanisms crucial to understanding how to prevent this event from occurring^[108]^. Trastuzumab is a monoclonal antibody that selectively targets the HER-2 receptor, an RTK overexpressed in approximately 20% of breast cancers^[109]^. By binding the extracellular domain (ECD) of HER2, trastuzumab blocks ligand-independent receptor dimerization, reduces downstream proliferative signaling (via PI3K/Akt and MAPK pathways), and enhances antibody-dependent cellular cytotoxicity (ADCC)^[109]^. Despite its efficacy, primary and acquired resistance to trastuzumab remain significant challenges. These resistance mechanisms include factors hindering trastuzumab binding to HER2; upregulation of HER2 downstream signaling pathways; and signaling through alternate pathways^[110-116]^.

As suspected, one of the more common mechanisms of acquired resistance is receptor modification. More specifically, this resistance is characterized by p95HER2 overexpression^[117]^. p95HER2 is an amino terminally truncated membrane-bound fragment that is generated from the cleavage of the ECD following ligand binding^[117]^. High expression levels of p95HER2 make the cells resistant to trastuzumab as it cannot bind to p95HER2 due to ECD loss, which, unsurprisingly, is correlated with distant metastases and resultant decreased survival^[117]^.

Upregulating downstream HER-2 signaling pathways is another mechanism in which BC can resist targeted therapy. Just as it is in other cancers, the RAS/Raf/MAPK and the PI3K/Akt cascades are the primary downstream signaling pathways in BC and play an important role in BC survival^[118]^. Specifically, loss-of-function PTEN deletions and activating mutations of PI3KCA, two of the most frequent genetic alterations in BC, cause increased activity of the PI3K/Akt pathway and can contribute to trastuzumab resistance^[118]^.

While HER-2 is a key mediator of cell survival, there are other receptors (EGFR/HER1, HER3, and HER4) within the HER family that also have the potential to form dimers and mediate signaling^[117]^. Therefore, even with successful trastuzumab binding and inhibition, crosstalk still allows for the activation of alternative signaling pathways such as ER, IGF-1R, and HER3 signaling pathways^[117]^. For instance, IGF-1R is crucial in resistance and overexpressed in approximately 43%-50% of primary BC^[117-119]^. Aberrant activation of IGF-1R has been shown to upregulate the expression of the p27Kip1 ubiquitin ligase, SKP2^[117-119]^. This leads to the degradation of the cyclin-dependent kinase inhibitor (CDKI) p27Kip1, causing a loss of growth arrest^[117-119]^. Another example is HER3 acting as a crucial cofactor for sustaining cell proliferation in HER-2-overexpressing cell lines^[120]^. The heterodimerization of HER2 with HER3 activates the PI3K/Akt signaling pathway through the phosphorylation of HER3 receptors at multiple tyrosine residues^[120]^. The c-MET receptor and its ligand, HGF, further contribute to trastuzumab resistance by inhibiting trastuzumab-mediated p27 induction and continued Akt activation^[120-122]^. The increased expression of the receptor tyrosine kinase Eph receptor A2 (EphA2) has also been implicated in both intrinsic and acquired trastuzumab resistance^[120-122]^.

In breast cancer, these alterations contribute to therapeutic resistance, especially in HER-2-positive subtypes treated with trastuzumab. The resistance mechanisms encompass not only direct alterations affecting HER2 but also downstream pathway modulations, further highlighting the complexities involved in treating resistant tumors^[122]^.

#### Prostate carcinoma and androgen receptor resistance

PCa is the most common cancer in men, and there will be nearly 35,250 PCa-related estimated deaths in 2024, ranking second in cancer-related deaths in the United States^[123-125]^. Although most patients present with localized disease, progression to metastatic disease and its management remain significant clinical challenges. Most patients with advanced disease progress to metastatic castration-resistant prostate cancer (mCRPC), associated with a median OS of 4-5 years^[126]^. The prostate epithelium, as well as the cancerous cells, express high levels of AR, which encodes the androgen receptor (AR), and this has been associated with hormonal dependency in PCa^[127]^. The shift between an AR signal mainly associated with epithelial growth and differentiation to signaling associated with indiscriminate growth in the cancer scenario is unclear; nonetheless, although this AR pathway is the cornerstone of current therapies, resistance arises in the context of androgen deprivation therapy (ADT) and androgen blockade^[128,129]^. It is vital to note that AR activity is necessary for tumor development and is the primary driver of disease progression to the castration-resistant phase during ADT. The AR predominantly functions as a transcription factor in normal prostate homeostasis^[130,131]^. Genes such as KLK3, encoding prostate-specific antigen (PSA), are direct transcriptional targets of the AR, and their expression is often used as a surrogate for AR activity in PCa research and clinical monitoring^[132]^. The disease state is established where AR primarily drives a growth-related genetic program. Significantly, ADT is associated with alterations in the AR pathway, especially regarding AR overexpression and/or post-translation modifications that can lead to therapy resistance through multiple mechanisms. In this regard, most described mechanisms leading to castration resistance are mediated by AR or its related axis.

In mCRPC, for instance, there are increased mutations, amplifications of, and gain-of-function of AR. Amplification of the AR has been identified in up to 20% of mCRPC patients and is associated with response to the low levels of circulating and/or intra-tumor androgens^[132]^. It should also be noted that AR amplification is a unique characteristic of prostate tumors that have been exposed to androgen deprivation, indicating that AR amplification is a consequence of hormone therapy^[133]^. Reportedly, 70% of mCRPC patients have amplification or alterations of regulators for AR transcription, such as FOXA1^[134]^. Some genes that repress AR pro-tumorigenic signaling, like tumor suppressors ZBTB16 and NCOR1, have inactivating mutations or deletions^[130,131]^. By contrast, for metastatic castration-sensitive PCa, follow-up targeted genetic studies in matched samples of patients who later displayed progression onto mCRPC have shown that AR is altered in only 2%-6%, suggesting an acquired role for AR amplifications and mutations in mCRPC.

There are also reports of enhancers and regulators that have the potential to increase the expression of the *AR* gene independently of AR locus amplification in response to ADT^[135]^. In metastatic PCa, ADT has been associated with a mechanism of increased sensitivity to circulating androgens. A substitution of valine with leucine at codon 89, which is associated with more aggressive and early-onset PCa, has also been associated with increased 5α-reductase levels in a subset of mCRPC patients, rendering this population with higher levels of DHT despite low circulating levels of testosterone^[135]^. The substitution of threonine with alanine at codon 877 on the ligand binding domain of the AR, as well as L701H, V715M, and W741C substitutions, are among point mutations identified in the *AR* gene that lead to increased AR activity in the presence of low levels of androgens as well as non-androgenic steroids, such as hydrocortisone and estradiol.

There are also several AR co-activators, such as SRC1, SRC2, SRC3, ARA70, and PIAS1, that can interact with the AR and subsequently enhance its activity^[136,137]^. The key AR regulators, TIF2 and SRC1, have been associated with higher expression levels following androgen deprivation and are overexpressed in mCRPC samples. For instance, TIF2 enhances the AR transcriptional activation in response to adrenal androgens (DHEA and androstenedione)^[138]^.

Identifying androgen receptor variants (AR-Vs) in tumors derived from mCRPC patients provides further mechanistic insight into the CRPC phenotype development^[139,140]^. AR-Vs are splice variants of AR that are constitutively active due to the loss of the C-terminal LBD. Significantly, treatment-induced AR amplification in CRPC may contribute to developing receptor variants. In a recent report, a 48-kb deletion in AR intron 1 was linked to the expression of the AR-V7/AR3 splice variant in the CWR-R1 cell line. After AR blockade with enzalutamide, the AR-V7/AR3-expressing clone was associated with tumor growth during ADT, providing mechanisms of AR splice variants in the pathogenesis of CRPC^[139,140]^.

#### Pancreatic cancer and resistance to KRAS-targeted therapies

To date, pancreatic cancer remains one of the most challenging malignancies to treat, with a 5-year survival rate approaching 13%. In PAAD, the KRAS mutation is understood to be an early driver of tumorigenesis, as it is mutated in around 90%-95% of cases^[141]^. These mutations usually occur at the G12 site, with the G12D and G12V mutations making up 39.2% and 32.5% of alterations, respectively, followed by G12R and G12C at 17.1% and 1.7%^[142]^.

The resultant effect is continual activation of KRAS downstream signaling, which promotes cancer cell growth and proliferation^[143]^. Aside from tumor intrinsic effects, mutant KRAS is also known to cultivate a proinflammatory and immunosuppressive environment by causing increased levels of TGFβ and IL-10 in the TME^[144]^. Additionally, *in vivo* studies demonstrated that KRAS mutation can drive highly mesenchymal subclones of PAAD cells that exhibit increased drug resistance^[145]^. Heterogeneity-driven resistance of PAAD is not just limited to varying states of EMT. The many parallel and redundant pathways branching off KRAS that drive cell survival and growth cause the effectiveness of KRAS inhibition to be highly context-dependent and subject to non-genetic acquired mechanisms of resistance.

The combination of all these effects from mutant KRAS primes the target to resist targeted therapies against it. This resistance is further compounded by the fibrotic nature and low vascularity of PAAD tumors, alongside a highly immunosuppressive TME^[146]^. This can create a formidable barrier against therapeutic interventions aimed at curbing tumor growth. However, the main reason underlying this tumor’s challenging target involves the dynamic and redundant signaling pathways activated by KRAS mutations. For instance, although ERK inhibition will reduce MAPK signaling in the short term, the drug-induced loss of the endogenous negative feedback capability eventually leads to MAPK activation rebounding to a new steady state, preventing a durable drug response^[147]^. A further factor limiting the effectiveness of inhibiting nonmutated proteins of the KRAS effector pathways is that therapies are not selective to mutant KRAS PAAD cells, substantially narrowing the therapeutic window due to toxic effects on normal physiological processes^[142]^.

#### OvCa and resistance to poly (ADP-ribose) polymerase inhibitors

Despite advances in understanding the biology underpinning OvCa in the last decade, with an annual burden of 300,000 new cases, it continues to be the major cause of gynecological cancer-related deaths globally and the eight-leading cause of cancer deaths in women^[148-150]^. In most patients, OvCa tends to be diagnosed at an advanced stage, which, in combination with its heterogeneous molecular makeup, lends to its high mortality rate^[151-153]^. In 2023, the United States had an estimated 13,270 OvCa-related deaths and 5-year survival outcomes of around 30%-50%, depending on the stage at diagnosis^[149]^. The most common histological subtype of epithelial OvCa is high-grade serous ovarian cancer (HGSOC), which accounts for 60%-80% of all cases^[148]^. The mainstay of treatment is cytoreductive surgical debulking and platinum- or taxane-based chemotherapeutic regiments. Maintenance therapy typically includes VEGF inhibitors such as bevacizumab or, more recently, poly (ADP-ribose) polymerase inhibitors (PARPi). Some strategies to overcome PARP inhibitor resistance have been suggested^[154-157]^.

PARPs are some of the best-known components of the DNA damage response (DDR)^[158]^. They detect single-strand breaks (SSBs) and recruit additional repair factors through poly-ADP ribosylation (PARylation) of target proteins, including chromatin-associated proteins^[158]^. By inhibiting PARP activity, PARPi prevent the repair of SSBs, leading to the accumulation of double-strand breaks and synthetic lethality in tumors with defective homologous recombination repair (HRR) (e.g., BRCA1/2-mutant cancers)^[158]^. HGSOC are under immense DNA replication stress and rely on DNA HRR via tumor suppressors such as *BRCA1* and *BRCA2* to maintain chromosomal stability. PARPi take advantage of this dependency by binding the active site of PARP, inhibiting its catalytic activity and trapping the PARP-DNA complex and destabilizing replication forks^[159,160]^. Initially approved for maintenance treatment of recurrent platinum-sensitive *BRCA1/2* mutant epithelial OvCa, PARPi have shown benefits beyond these indications for HGSOC^[161]^. Despite all these advances, treatment-induced acquired resistance arises, which erodes the efficacy of these agents. Acquired resistance can be attributed to increased drug efflux from overexpression of multidrug efflux proteins, upregulation of survival pathways, and downregulation of DNA damage repair mechanisms^[162]^. Efflux proteins utilize ATP pumps to actively pump out the drug molecules before they can exert their effects intracellularly. The most notable examples are the adenosine triphosphate-binding cassette superfamily^[163]^.

Activation of alternate oncogenic pathways such as PI3K/Akt/mTOR and RAF/MEK have also been described as potential mechanisms for resistance to PARPi due to the PARP-PI3K-Akt crosstalk with HRR. Preclinical studies show increased activation of the PI3K-Akt pathway in the setting of PARPi, inducing apoptosis resistance and limiting the cytostatic efficacy of PARPi^[164]^. Due to its regulation of key cellular processes including metabolism, motility, and growth, it stands to reason why PI3K/Akt/mTOR dysregulation leads to the aberrant proliferation of cancer cells.

A wide array of genomic alterations has been described involving *PIK3CA*, *PTEN*, *AKT*, *TSC1*, *LKB1*, and *MTOR*^[165]^. Early phase studies showed promising activity using a combination of a pan-PI3K inhibitor (buparlisib) and MEK inhibitor (trametinib) to overcome resistance in patients with low-grade serous OvCa with refractory *RAS* or *RAF* mutations and yielded an ORR of 29%. However, a significant portion of the patients had grade 3/4 AEs, including transaminitis, creatinine kinase elevation, and rash, which might limit the future utility of these agents in combination therapy^[165-167]^.

A potential clinical predictive marker for PARPi resistance may be sensitivity to platinum-based chemotherapy. In HGSOC, both drug classes target DNA repair via pathways that likely share resistance commonalities such as secondary *BRCA* reversion mutations, loss of *53BP1*, and replication fork protection^[168-170]^. Resistance to PARPi gives rise to dependence on other DNA repair pathways, leading to additional therapeutic opportunities. Since *TP53* is almost ubiquitously lost in HGSOC, it points toward increased oncogenic stress. Ataxia telangiectasia and Rad3-related kinase (ATR) is a well-known regulator of cell death and controls cell cycle arrest from the S to G2 phases. In cells with *TP53* mutation, ATR promotes checkpoint-defective cells, and the inhibition of this target may prove synthetically lethal^[159,171,172]^. Overcoming acquired resistance in OvCa will take combination therapy for continued improvements. The combination of PARPi and ATRi has shown a synergistic effect in terms of DNA damage and durable tumor regression. The CAPRI trial evaluated the combination of Olaparib and ceralasertib in platinum-sensitive HR-deficient HGSOC, which progressed on prior PARPi and had an ORR of 46% (*n* = 6) and median PFS of 7.5 months with acceptable toxicity^[172]^. Other combinations such as ATRi plus gemcitabine have also shown clinical efficacy in platinum-resistant HGSOC^[173]^. Combinations of PARPi and PI3K/Akt inhibitors are also currently being clinically evaluated^[174,175]^. Alternate treatment paradigms are also being evaluated, including ADC such as mirvetuximab^[176]^, next-generation PARPi with higher selectivity, PDL1/PD1 inhibitors, and other additional promising targets such as DNA polymerase theta (POLθ)^[177,178]^ and novel oncogenes such as UBE2S^[179]^.

#### Glioblastoma and treatment resistance

WHO grade IV, IDH-WT astrocytoma (GBM) is the most common aggressive form of primary brain cancer^[180]^. The development of targeted therapies for GBM has been significantly challenged by the complex mechanisms of resistance inherent to the tumor’s biology.

One of the primary treatment challenges in treating GBM is the blood-brain barrier (BBB), a network of endothelial cells, pericytes, and astrocytic foot processes that prevent many therapeutic compounds from entering the brain^[181,182]^. Even though the integrity of this network is relatively compromised in GBM, the aberrant features of GBM allow for the formation of the blood-brain tumor barrier (BBTB)^[181,182]^. While more permeable to circulating nutrients that the tumor needs, drug delivery remains an issue owing to an upregulation of efflux transporters such as MDR1 and P-glycoprotein that expel drug molecules^[183-185]^. Mechanical disruption methods like osmotic disruption and focused ultrasound have been attempted to enhance drug delivery through these barriers; however, these strategies have had limited success due to ineffectiveness or an unacceptable level of toxicity^[186,187]^. To date, nanocarriers and peptide-based drug delivery methods are being explored to improve penetration across this barrier, but more research is needed to validate the long-term efficacy and safety^[188,189]^.

A major obstacle in treating GBM is the profound intra- and intergenetic heterogeneity. Because of this, effective targeted therapies are difficult to develop as treatments may only be effective against a specific subtype of cells, leading to recurrence following treatment as the resistant (non-targeted) cell populations continue to proliferate^[190-192]^. While there are several signaling pathways that are implicated in GBM pathogenesis, the Cancer Genome Atlas (TCGA) identified the RTK/RAS/PI3K, p53, and Rb pathways as foundational to GBM development^[193]^. As such, genetic alterations in these pathways contribute significantly to treatment resistance as crosstalk allows for the upregulation of downstream elements even in the presence of highly specific inhibitors. This is evident from the limited success of EGFR inhibitors despite the high prevalence of EGFR amplifications (including the unique variant EGFRvIII) in GBM^[194,195]^. In a similar fashion, the PI3K/Akt/mTOR pathway is also frequently activated in GBM, but is difficult to target due to redundancy with other signaling pathways and poor penetration across the BBB^[196]^. These pathways do not function in isolation; instead, they interact and compensate for one another.

Along with redundancy, these tumors also have developed specific resistance mechanisms to various agents. The most well-known is the developed resistance to the alkylating agent temozolomide (TMZ). TMZ acts by methylating guanine nucleosides, which nicks DNA and leads to apoptosis due to the inability to repair the damaged DNA^[197]^. However, when the DNA repair enzyme O6-methylguanine (O6-MeG)-DNA methyltransferase (MGMT) is active, it specifically reverses this action and renders TMZ ineffective^[191,198]^.

Epigenetic silencing plays a role in keeping this protein at bay; however, once TMZ therapy is initiated, the tumor undergoes genetic remodeling, leaving the MGMT promoter region unmethylated and promoting tumoral resistance^[191,198]^. While this mechanism is the main cause of drug resistance in the therapy of recurrent GBM, other mechanisms such as MMR deficiencies can also contribute to TMZ resistance. When the MGMT enzyme is absent during DNA replication, the DNA polymerase enzyme incorrectly inserts thymine at the site of O6-MeG, which initiates the formation of the MMR complex^[199,200]^. Although not directly cytotoxic, the O6-MeG lesion induces cellular toxicity due to MMR complex recognition and processing, triggering cell death either by futile cycling or direct signaling^[201]^.

Glioma-initiating cells (GICs) can also play a role in resistance in GBM. These cells reside in the hypoxic core of these tumors, where they contribute to resistance to radiotherapy via the kinases Chk1 and Chk2^[202,203]^. These kinases are activated in response to DNA damage signals, and their activity results in reduced sensitivity to radiation therapy^[204,205]^. GICs also express ABC at higher rates, which function as another obstacle for different therapies. Specifically, in GICs, hypoxia leads to upregulation of ABCC1 and ABCB1, promoting resistance to targeted therapies^[206]^. Hypoxia can also complicate matters as it both promotes the growth of GICs and upregulates hypoxia-inducible factor 1 (HIF-1), which has many downstream tumorigenic pathways^[206,207]^. Hypoxia-induced autophagy is a mechanism GICs use to survive under stress, which doubles as a resistance mechanism to these tumors. There are promising results showing that by inhibiting this specific autophagy in GICs with agents like chloroquine, sensitivity to TMZ is increased^[206,208-210]^.

## EMERGING STRATEGIES TO OVERCOME RESISTANCE

Throughout this discussion, we have delved into the myriad ways through which both solid and hematologic tumors develop resistance to targeted therapies. It is clear there are considerable overlaps in the adaptations these tumors employ, shedding light on the intricate struggle against cancer resistance. Equally as important, however, is looking at the various strategies employed to overcome these hurdles.

### Synthetic lethality and exploiting genetic vulnerabilities

**Figure 2:**
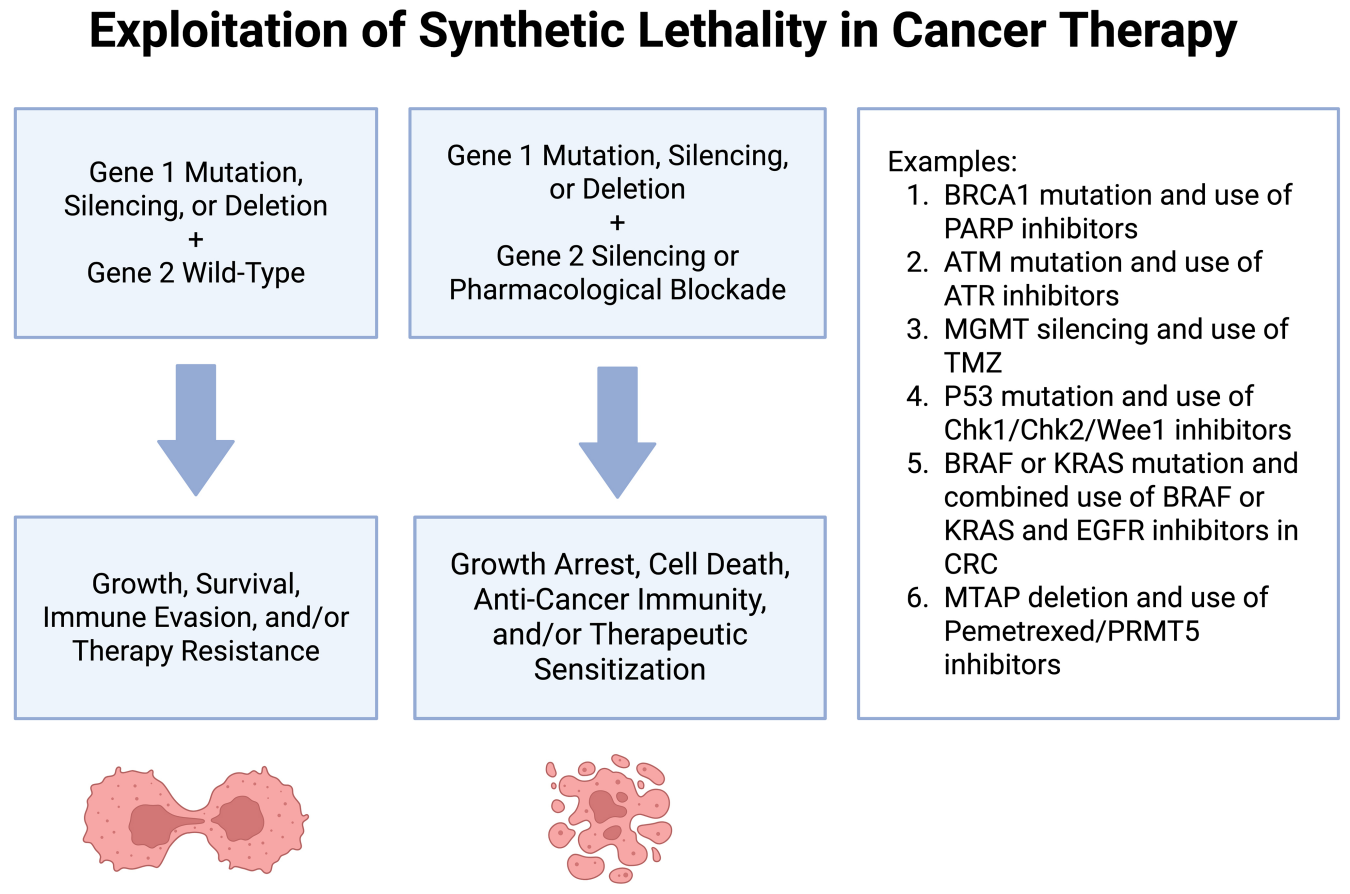
Exploitation of synthetic lethality in cancer therapy. The concept of synthetic lethality is represented along with examples of its clinical or experimental use in cancer therapy. Created in BioRender. Purcell, C. (2025) https://BioRender.com/hbbdh35.

Synthetic lethality [Figure 2] explains a scenario where alterations in two specific genes simultaneously lead to cell death^[211]^. Cancer cells possessing a mutation in only one of a particular pair of genes may rely on the unmutated partner gene to survive. Targeting and disrupting the activity of this unmutated partner gene could result in the death of the cancer cells. Investigating the concept of synthetic lethality offers insights into gene function and aids in the creation of novel cancer therapies. For example, in NSCLC, overcoming resistance to EGFRi can be approached through synthetic lethality by targeting the NF-κB pathway^[212,213]^. Some studies highlight the role of NF-κB in promoting resistance to EGFRi treatment, showing that silencing specific genes related to the NF-κB and Fas death receptor signaling pathways can sensitize NSCLC cells to erlotinib^[212,213]^. The introduction of PBS-1068, an inhibitor targeting the RELA subunit of NF-κB, demonstrates promising results in not only enhancing the response to erlotinib but also inducing apoptosis in both intrinsic and acquired EGFRi resistance scenarios^[213]^.

### Adaptive and dynamic therapy approaches

Overcoming acquired resistance to MTTs will require a paradigm shift from linear, single-agent treatments to adaptive, dynamic therapeutic strategies. One promising avenue is the application of adaptive therapy, which intentionally cycles drugs or adjusts dosing to prevent resistant clones from gaining dominance^[214]^. This approach leverages the concept of competitive suppression, wherein sensitive tumor cells keep resistant clones in check, effectively turning tumor heterogeneity into a therapeutic advantage (CUSP9 protocol)^[215,216]^.

Simultaneously, advances in multiomic profiling - integrating genomics, transcriptomics, epigenomics, and proteomics - will allow clinicians to map the evolving resistance landscape in real time, guiding treatment modifications based on the tumor’s shifting vulnerabilities^[217]^. This personalized “resistance fingerprinting” could transform therapeutic decision making, shifting from reactive to proactive resistance management.

### Targeting TME and non-oncogene dependencies

Beyond established targets, the future will also emphasize non-oncogene dependencies - exploiting metabolic bottlenecks, stress response pathways, and epigenetic vulnerabilities that emerge specifically in resistant cells^[217]^. Drugs targeting CAF signaling, exosome biogenesis, or tumor metabolic crosstalk may work synergistically with targeted therapies to block resistance-enabling interactions within the TME^[218,219]^.

One example of targeting the TME is the inhibition of Tie2-expressing monocytes, which has been shown to impair tumor angiogenesis and progression^[220]^. Another approach is the use of tasquinimod, which inhibits MDSCs and angiogenesis within the TME, demonstrating the potential of disrupting non-oncogenic pathways to counteract resistance^[221]^.

### Next-generation molecularly targeted agents

Next-generation molecularly targeted agents will be engineered not only to inhibit driver pathways but also to anticipate common resistance mutations, incorporating elements of induced synthetic lethality to exploit vulnerabilities that arise only after resistance emerges. These future agents will likely combine multi-target inhibition with context-dependent activation to selectively disable both primary oncogenic drivers and the adaptive pathways that resistant cells rely on for survival^[222,223]^.

A prime example of next-generation targeted therapy is the development of Hedgehog pathway inhibitors such as vismodegib and sonidegib, which are designed to block a key signaling pathway implicated in tumor progression and therapy resistance^[224]^.

Together, these approaches represent a necessary evolution from static, pathway-centered therapy to dynamic, system-aware intervention, where therapy itself adapts in parallel with the evolving tumor.

## CONCLUSION

Although much progress has been made in unraveling tumor biology, developing models, analyzing human tumor tissues at high resolution, defining cancer hallmarks, and devising novel therapeutic strategies, significant challenges remain in prolonging survival or curing more cancers. In the United States alone, each year, there are more than 600,000 cancer-related deaths despite all the advancements in research and treatment^[1]^. The complexity of tumor and host heterogeneity, along with the evolutionary dynamics of tumor adaptation, presents an ongoing challenge in the field. Tumors constantly evolve under selection pressure imposed by therapy, leading to resistance mechanisms that necessitate the development of multifaceted approaches^[225]^.

The interplay between tumor microenvironmental factors, immune evasion, and metabolic reprogramming further complicates treatment strategies. Understanding these ecological and evolutionary dynamics not only presents obstacles but also provides opportunities for novel therapeutic interventions^[226,227]^. Another significant barrier remains the toxicity of cancer therapies, both in physiological and financial terms, which limit accessibility and tolerability for many patients^[228]^.

Future research directions must integrate a holistic approach that acknowledges the complexity of the TME, the evolving resistance mechanisms, and the heterogeneity of cancer^[228]^. Advancements in precision oncology will continue to drive highly individualized treatment strategies, leveraging adaptive resistance-targeting therapies such as synthetic lethality-based interventions and real-time multiomic profiling. The next phase of drug development must incorporate therapies that anticipate resistance mutations and dynamically adapt to the evolving tumor landscape^[229,230]^.

Moving forward, a dynamic and system-aware treatment paradigm is required - one that embraces continuous adaptation and innovation in oncology. This will necessitate interdisciplinary collaborations, novel computational and genomic methodologies, and the sustained refinement of precision medicine strategies to ultimately improve patient outcomes in the battle against acquired resistance.

## DECLARATIONS

### Copyright

© The Author(s) 2025.
